# Fully epitaxial *C*1_b_-type NiMnSb half-Heusler alloy films for current-perpendicular-to-plane giant magnetoresistance devices with a Ag spacer

**DOI:** 10.1038/srep18387

**Published:** 2015-12-17

**Authors:** Zhenchao Wen, Takahide Kubota, Tatsuya Yamamoto, Koki Takanashi

**Affiliations:** 1 Institute for Materials Research, Tohoku University, Sendai 980-8577, Japan

## Abstract

Remarkable magnetic and spin-dependent transport properties arise from well-designed spintronic materials and heterostructures. Half-metallic Heusler alloys with high spin polarization exhibit properties that are particularly advantageous for the development of high-performance spintronic devices. Here, we report fully (001)-epitaxial growth of a high-quality half-metallic NiMnSb half-Heusler alloy films, and their application to current-perpendicular-to-plane giant magnetoresistance (CPP-GMR) devices with Ag spacer layers. Fully (001)-oriented NiMnSb epitaxial films with very flat surface and high magnetization were prepared on Cr/Ag-buffered MgO(001) single crystalline substrates by changing the substrate temperature. Epitaxial CPP-GMR devices using the NiMnSb films and a Ag spacer were fabricated, and room-temperature (RT) CPP-GMR ratios for the *C*1_b_-type half-Heusler alloy were determined for the first time. A CPP-GMR ratio of 8% (21%) at RT (4.2 K) was achieved in the fully epitaxial NiMnSb/Ag/NiMnSb structures. Furthermore, negative anisotropic magnetoresistance (AMR) ratio and small discrepancy of the AMR amplitudes between RT and 10 K were observed in a single epitaxial NiMnSb film, indicating robust bulk half metallicity against thermal fluctuation in the half-Heusler compound. The modest CPP-GMR ratios could be attributed to interface effects between NiMnSb and Ag. This work provides a pathway for engineering a new class of ordered alloy materials with particular emphasis on spintronics.

Engineering of advanced half-metallic materials is of particular importance for achieving ideal magnetic and spin-dependent transport properties for a variety of significant spintronic applications, because 100% spin polarization of the conductive electrons at the Fermi level is theoretically predicted for these series of materials^1^,^2^,^3^,^4^. Heusler alloys, a type of half-metallic materials, have properties that are particularly advantageous to achieving half-metallicity at room temperature (RT)^3^,^5^,^6^, and have been attracting extensive interest for the development of next-generation spintronic devices, such as magnetoresistive random access memories (MRAMs)^7^,^8^,^9^, current-perpendicular-to-plane giant magnetoresistance (CPP-GMR) read heads for hard disk drives (HDDs)^10^,^11^,^12^,^13^, spin torque oscillators (STO)^14^,^15^,^16^, and spin transistors^17^,^18^. Half-metallic Heusler alloys are classified into full- and half-Heusler compounds with the chemical formula of X_2_YZ in the *L*2_1_ structure and XYZ in the *C*1_b_ structure (X and Y: transition metals; Z: non-magnetic element)^6^, as shown in Fig. 1. Recently, cobalt-based full-Heusler alloys were proposed as a candidate material for high-output CPP-GMR effect, which is a key technology for next-generation HDDs with an areal density of more than 2 Tb/inch^2^,^7^,^19^,^20^. CPP-GMR junctions have been fabricated using several full-Heusler compounds, such as Co_2_Fe(Al-Si)^10^, Co_2_MnSi^11^, Co_2_(Fe-Mn)Si^12^, and Co_2_Fe(Ge-Ga)^13^, and these studies promoted the further development of Heusler alloys for practical applications. However, despite these developments, it remains difficult to overcome the spin-transfer torque and Joule heating problems associated with realizing ultra-high density HDDs. Therefore, advanced materials with high-performance half-metallicity are desired for further improvement of CPP-GMR junctions.

The bandgap in half-metallic Heusler alloys originates from the strong *d*-band hybridization of the two transition metals^21^. For full-Heusler alloys, the bandgap is dominated by the *d*-band hybridization between elements X (X-X hybridization). The antibonding states formed by X–X hybridization cannot couple with the Y degenerates, resulting in a small gap across the Fermi level (*E*_F_). For half-Heusler alloys, however, the origin of the bandgap is the hybridized states of elements X and Y, which can form a very large bandgap between the bonding (*t*_2g_) and antibonding (*e*_g_) degenerates. Figure 1 illustrates a comparison of crystalline cell structures and the electronic band structure near the Fermi level between *L*2_1_-type full-Heusler and *C*1_b_-type half-Heusler alloys. Based on the origin of their bandgaps, half-Heusler alloys with a large bandgap (e.g. 0.5 eV for NiMnSb and 1 eV for CoMnSb)^21^ are promising for the development of high-performance magnetoresistive devices owing to the suppression of thermal activations of the electrons above the bandgap. Nevertheless, previous studies have demonstrated that the half-metallic properties of half-Heusler alloys can be very easily degraded by disordering and the presence of defects and/or surface segregation and termination atoms^22^,^23^,^24^, and a few percent CPP-GMR ratio was only observed at low temperature (4.2 K) in a polycrystalline NiMnSb/Cu/NiFe system^25^. This indicates that a precise control over the ordering and structure of half-Heusler alloys is highly desired.

In this work, we open a new field in Heusler spintronics, i.e. half-Heusler compounds for CPP-GMR devices, and room-temperature CPP-GMR ratios are achieved in fully epitaxial NiMnSb/Ag/NiMnSb structures for the first time. High-quality half-Heusler NiMnSb films with an almost perfectly stoichiometric composition were fabricated using a co-sputtering method. Fully (001)-orientation epitaxy NiMnSb alloy films with high magnetization and a very flat surface were achieved on Cr/Ag-buffered MgO(001) single crystalline substrates by optimizing the substrate temperature (*T*_substrate_). CPP-GMR effect in the NiMnSb/Ag/NiMnSb structures was investigated. Anisotropic magnetoresistance (AMR) measurement in a single epitaxial NiMnSb film indicates robust half metallicity against thermal fluctuation in the half-Heusler compound.

## Results

### Structural and magnetic properties

**Figure 2:**
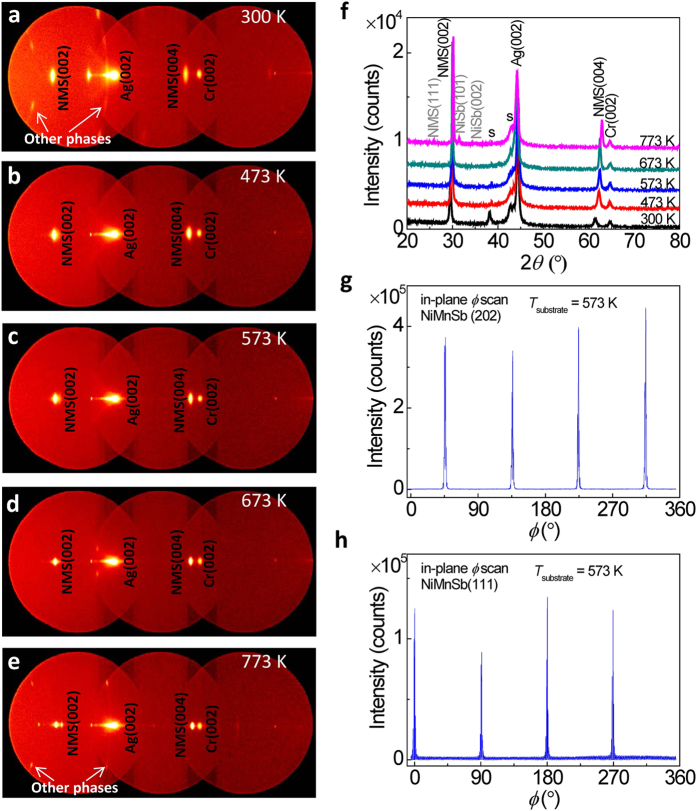
Epitaxial structure analyses of NiMnSb films by XRD patterns. (**a**–**e**) Two-dimensional out-of-plane (2*θ*-scan) XRD patterns for 50-nm-thick NiMnSb(NMS) films deposited on Cr(20 nm)/Ag(40 nm)-buffered MgO(001) single crystalline substrates at *T*_substrate_ varying from 300 to 773 K. (**f)** Extracted out-of-plane XRD data in a zero-dimensional region for the samples. (**g**,**h**) In-plane (*ϕ*-scan) XRD patterns of the NiMnSb(202) plane and NiMnSb(111) plane for the sample with the structure of MgO(001)-substrate//Cr(20)/Ag(40)/NiMnSb(50) (unit: nm) at *T*_substrate_ = 573 K, which were obtained by tilting the sample plane to *χ* = 45° and 54.7°, respectively. The intensity is shown on a linear scale.

Half-Heusler NiMnSb films were formed by a co-sputtering method using Ni and MnSb targets. After optimizing the sputtering rate of both the targets, the composition of the deposited NiMnSb films was confirmed to be Ni_1.01 ± 0.02_Mn_0.98 ± 0.02_Sb_1.01 ± 0.02_ by inductively coupled plasma (ICP) analysis, which is an almost ideal stoichiometric composition for the half-Heusler compound. We deposited the films on Cr(20 nm)/Ag(40 nm)-buffered MgO(001) single crystalline substrates at varied *T*_substrate_ ranging from RT to 773 K. The structural properties were characterized by two dimensional (2D) out-of-plane (2*θ*-scan) X-ray diffraction (XRD) patterns, as shown in Fig. 2a–f. For the as-deposited NiMnSb film at RT, the out-of-plane diffraction peaks from the NiMnSb(002) and (004) super lattices are clearly observed in addition to the Cr(002) and Ag(002) peaks from the buffer layers, while peaks corresponding to other phases and/or orientations of the NiMnSb layer also appear, as pointed out by the arrows in Fig. 2a. When the *T*_substrate_ increases to 473−673 K, the single phase and (001)-orientated peaks of NiMnSb can be seen in the deposited films. At *T*_substrate_ = 773 K, however, multi-phases and other orientations appear again, i.e. NiMnSb(111), NiSb(101) and NiSb(002), as shown in Fig. 2e. We extracted the 2D out-of-plane XRD data in a zero-dimensional diffraction region in Fig. 2f, where the phases of NiSb and NiMnSb(111) orientation are indicated. In order to confirm the *C*1_b_ structure and epitaxial growth of NiMnSb films, out-of-plane and in-plane (*ϕ*-scan) XRD patterns of the NiMnSb(202) and NiMnSb(111) planes were investigated by tilting the sample plane to *χ* = 45° and 54.7°, respectively. For the NiMnSb sample deposited at 573 K, the (202) and (111) peaks are clearly observed in Figure S1 (Supplementary Information), consistent with the *C*1_b_ structure. Figures 2g,h show typical in-plane (*ϕ*-scan) XRD patterns for the sample in which the 4-fold peaks are observed for both planes. This indicates that the NiMnSb film has a 4-fold symmetry and was epitaxially grown in the engineered structure of MgO(001)-substrate//Cr(20)/Ag(40)/NiMnSb(50) (unit: nm).

**Figure 3:**
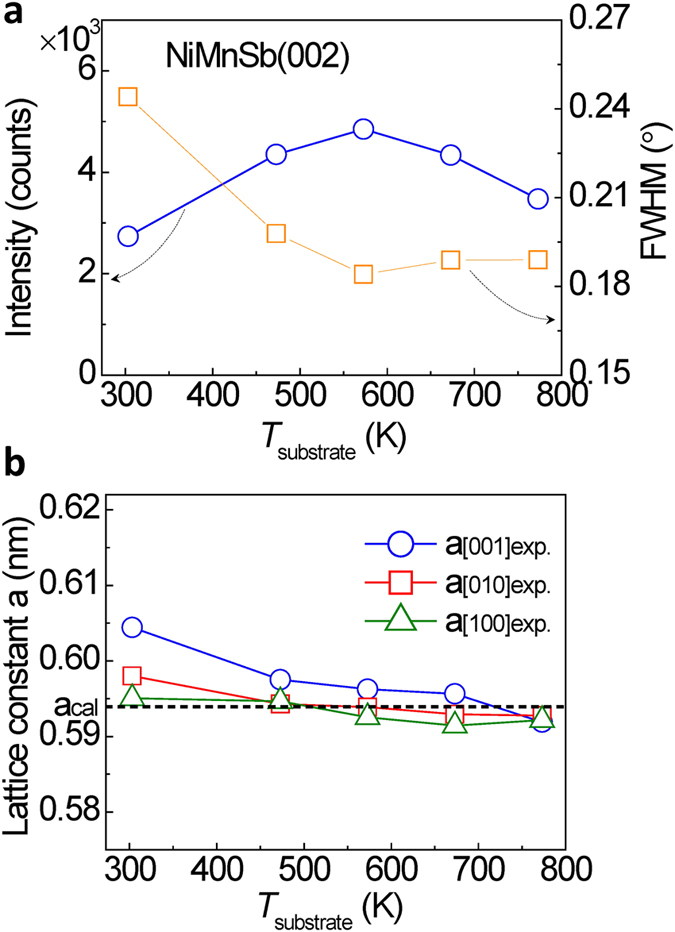
The dependence of NiMnSb crystal structures on *T*_substrate_. (**a)** Integrated intensity and full width at half maximum (FWHM) of NiMnSb(002) peaks as a function of *T*_substrate_ for the samples with the structure of MgO(001)-substrate//Cr(20)/Ag(40)/NiMnSb(50) (unit: nm). (**b**) The *T*_substrate_ dependence of the lattice constants of the NiMnSb films, derived from the XRD data of NiMnSb(002), (202), and (111) peaks.

Figure 3a shows the integrated intensity and the full width at half maximum (FWHM) of the NiMnSb(002) peaks as a function of *T*_substrate_ for the samples with the structure of MgO(001)-substrate//Cr(20)/Ag(40)/NiMnSb(50) (unit: nm). With increasing *T*_substrate_, the intensity of the peaks initially increases for the samples owing to the improved ordering and the (001)-epitaxy of the NiMnSb layers, and it reaches a maximum at *T*_substrate_ = 573 K. Furthermore, in accordance with the maximum in the peak intensity, a minimum in the FWHM was observed at *T*_substrate_ = 573 K, indicating that optimum crystalline quality was achieved at *T*_substrate_ = 573 K. The reduction in the peak intensity at a higher *T*_substrate_ may be caused by the generation of other phases. Figure 3b shows the *T*_substrate_ dependence of the in-plane and out-of-plane lattice constants of the NiMnSb films, derived from the 2*θ* positions of the NiMnSb(002), (202), and (111) peaks in the XRD patterns. With increasing *T*_substrate_, all the experimental lattice constants of *a*_[001]exp._, *a*_[010]exp._, and *a*_[100]exp._ monotonically decrease. The decrease in lattice constants may be explained by the increase of *C*1_b_ ordering of NiMnSb films as indicated in XRD results when the substrate temperature increases to the optimized value. With the *C*1_b_ structure enhancing, unoccupied Ni sites (vacancy sites) increase in the NiMnSb films, which results in the reduction in the lattice constants. However, when the substrate temperature further increases to a higher value than the optimized one, the other phases (such as NiSb) appear as observed in XRD results, which causes the Mn substitution for Sb or/and Ni sites in the NiMnSb films. Especially, the radius of Mn ion is much smaller than that of Sb, resulting in a further reduction in the lattice constants with increasing substrate temperature. Although the out-of-plane lattice *a*_[001]exp._ shows a slightly higher value than the others, the deviation between the observed lattice constants and the theoretical ones (*a*_cal._) is smaller than 0.4% at *T*_substrate_ = 573 K. This result suggests that an excellent (001)-epitaxy of NiMnSb films was successfully achieved on the Cr(20 nm)/Ag(40 nm)-buffered MgO(001) substrates without losing the half-metallicity^26^.

**Figure 4:**
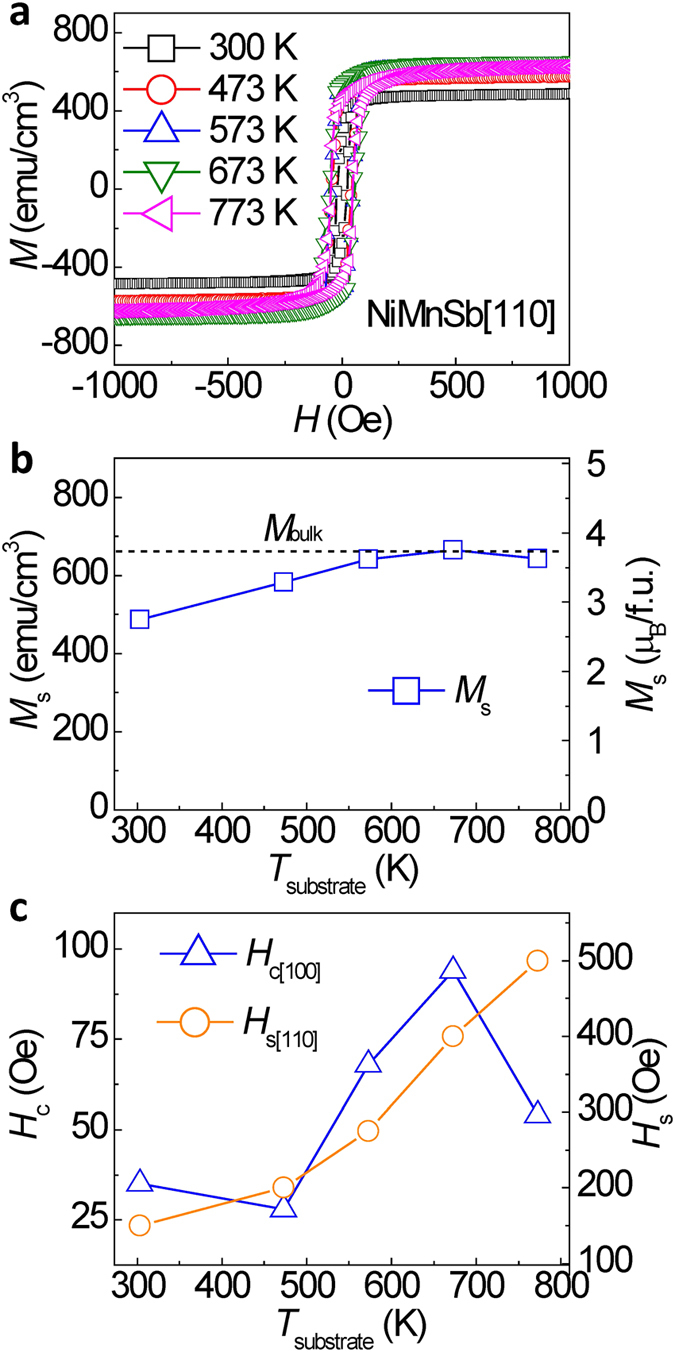
Magnetic properties of epitaxial NiMnSb films. (**a)** Magnetic hysteresis loops of epitaxial NiMnSb(50 nm) films with varying deposition temperature on the Cr/Ag-buffered MgO(001) substrates. (**b**) *T*_substrate_ dependence of saturation magnetization *M*_s_. (**c**) Coercivity *H*_c[100]_ and saturation magnetic field *H*_s[110]_ as a function of *T*_substrate_ for the NiMnSb films.

The magnetic properties of NiMnSb epitaxial films deposited at different *T*_substrate_ were investigated. Figure 4a shows the magnetic hysteresis (*M*-*H*) loops for 50-nm-thick NiMnSb films on the Cr/Ag-buffered MgO(001) substrates, which were measured along the NiMnSb[110] crystalline orientation. The saturation magnetization (*M*_s_) increases with increasing *T*_substrate_ to 673 K, and then decreases at 773 K, as shown in Fig. 4b. The increase in *M*_s_ is attributed to the increased order in the structure at a high temperature, while the reduction at 773 K could be due to the appearance of other phases and orientations in the NiMnSb films, as demonstrated in the XRD patterns. An optimum *M*_s_ value of ~3.7 *μ*_B_/*f*.*u*. was achieved at *T*_substrate_ = 573−673 K, which is comparable to the saturation magnetization (*M*_bulk_) of bulk NiMnSb at RT^27^,^28^. Figure 4c shows the easy-axis coercivity (*H*_c[100]_) and hard-axis saturation field (*H*_s[110]_) as a function of *T*_substrate_ for the NiMnSb films along the NiMnSb[100] (easy axis) and NiMnSb[110] (hard axis) crystalline orientations, respectively. Both *H*_c[100]_ and *H*_s[110]_ increase with increasing *T*_substrate_ for temperatures ranging between 200 and 673 K, indicating an improvement in magnetic anisotropy. The trend deviation of *H*_s[110]_ for the as-deposited sample and for that deposited at *T*_substrate_ = 773 K could be due to the multi phases and/or the poor epitaxy of the NiMnSb film as observed in XRD patterns. Further investigation on the mechanism of magnetic anisotropy is required.

**Figure 5:**
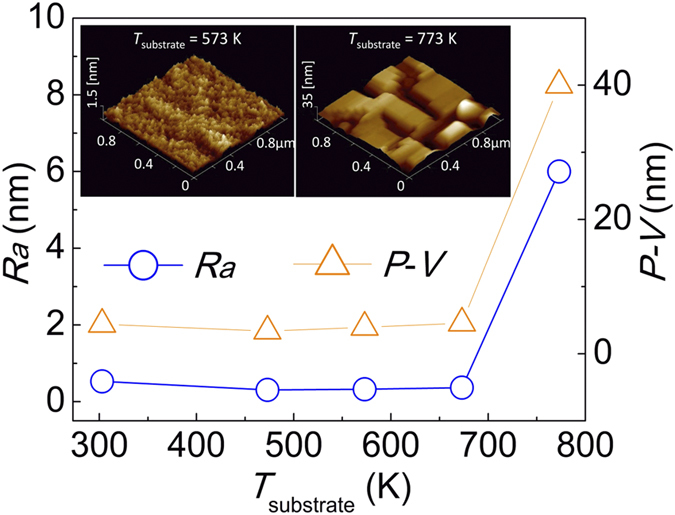
Surface morphology of NiMnSb half-Heusler alloy films on Cr/Ag-buffered MgO(001) single crystalline substrates as a function of *T*_substrate_. The NiMnSb films were 50-nm-thick and deposited at *T*_substrate_. Inset: AFM images of the surfaces of NiMnSb films deposited at 573 K and 773 K, respectively.

For stacking GMR multilayers using the fully (001)-epitaxial NiMnSb films, the *T*_substrate_ dependence of the surface morphology of NiMnSb films was investigated by atomic force microscopy (AFM). Figure 5 shows the average surface roughness (*R*_*a*_) and peak-to-valley (*P*-*V*) value as a function of *T*_substrate_ for 50-nm-thick NiMnSb films capped by 2-nm-thick Ag layers on Cr(20 nm)/Ag(40 nm)-buffered MgO(001) single crystalline substrates. On increasing the *T*_substrate_ to 573 K, the surface of the deposited NiMnSb film becomes slightly flatter than that of the sample deposited at RT. An optimally flat surface with *R*_*a*_ ∼ 0.35 nm and *P*-*V *∼ 3.9 nm was obtained at *T*_substrate_ = 573 K, and the high-quality surface without segregation is shown in the inset of Fig. 5. However, when the *T*_substrate_ is higher than 673 K, an abrupt increase in the surface roughness is observed. As shown in the inset of Fig. 5, for the sample deposited at 773 K, we can see the three-dimensional growth of square islands, which could be attributed to the nucleation and growth thermodynamics at a high temperature^24^. These results indicate that *T*_substrate_ ranging from 473 to 673 K is feasible for stacking whole CPP-GMR multilayers.

### Magneto-electrical transport properties

**Figure 6:**
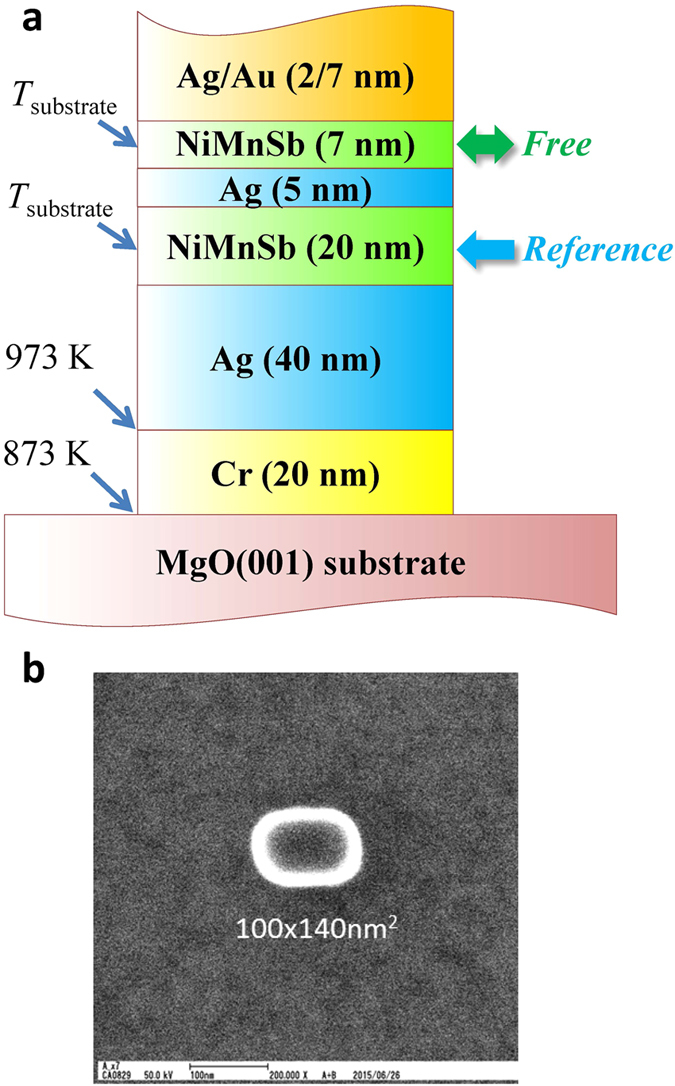
Multilayer structure and dimension of nanopatterned CPP-GMR devices. (**a**) Schematic of the structure of fully epitaxial NiMnSb/Ag/NiMnSb CPP-GMR stacks. The deposition condition of each layer is also shown, and the reference (free) NiMnSb layer was designed to be 20(7)-nm-thick. (**b**) SEM image of a typical CPP-GMR nanopillar.

Based on the above structural and magnetic investigations, whole CPP-GMR stacks with the structure of MgO(001)-substrate/Cr(20)/Ag(40)/NiMnSb(20)/Ag(5)/NiMnSb(7)/Ag(2)/Au(7) (unit: nm) were deposited by varying *T*_substrate_ (=473, 573, or 673 K), for the growth of NiMnSb layers. Figure 6a shows a schematic of the fully epitaxial CPP-GMR multilayers. For the whole stack, the XRD patterns show only single phase and (001)-orientation peaks, as shown in Figure S2 (Supplementary Information), indicating a uniform structure between the bottom and top NiMnSb layers because both of them were deposited upon the Ag(001) layer under the same conditions. The multilayer films were then nano-patterned into rectangular pillars with designed sizes ranging from 50 × 100 nm^2^ to 400 × 800 nm^2^ for the measurement of magneto-electrical transport properties. Figure 6b shows the scanning electron microscopy (SEM) image of a typical CPP-GMR nanopillar with a minimum dimension of 100 × 140 nm^2^.

**Figure 7:**
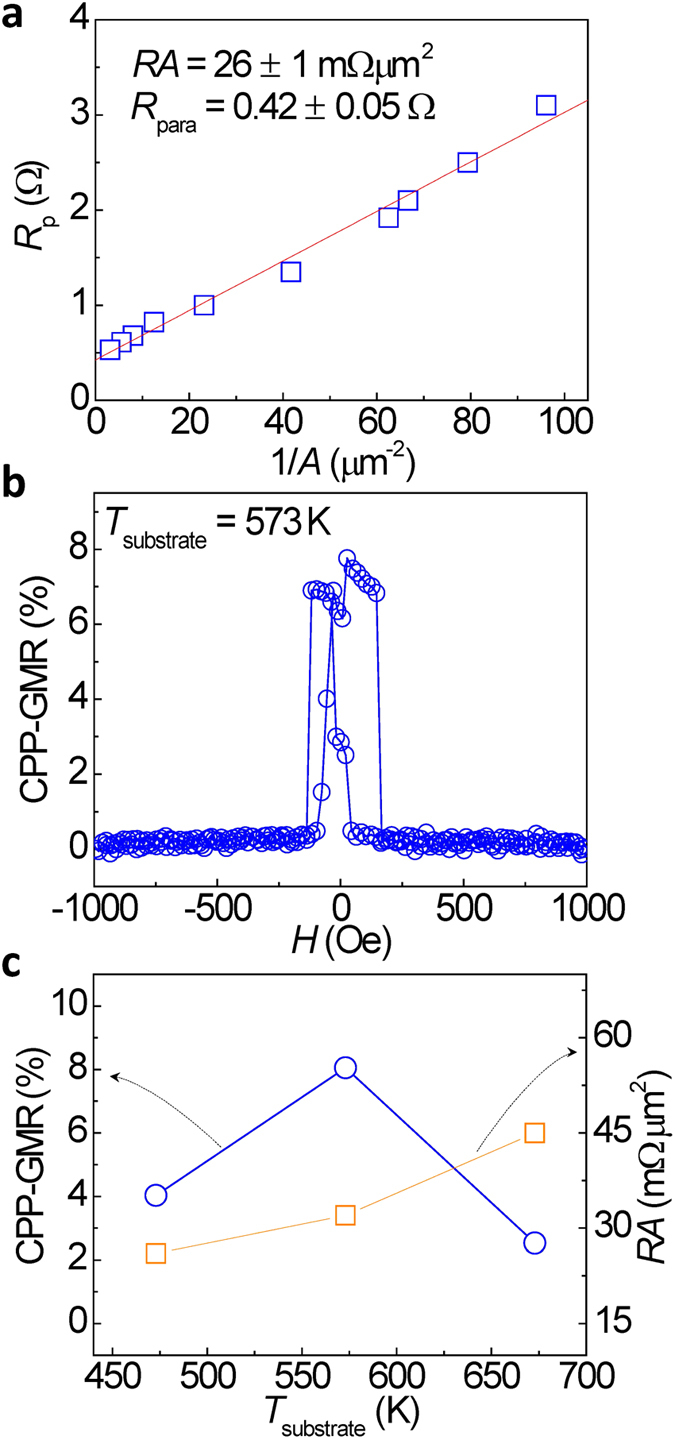
Magnetic transport properties of epitaxial NiMnSb/Ag/NiMnSb CPP-GMR devices at RT. (**a)** The dependence of junction resistance *R*_p_ on the inverse of the junction area (1/*A*) at the parallel magnetization configuration for patterned CPP-GMR devices with NiMnSb layers deposited at *T*_substrate_ = 573 K. (**b**) MR curves for a typical CPP-GMR nanopillar. (**c**) CPP-GMR ratio and *RA* value for CPP-GMR devices in which NiMnSb layers were deposited at *T*_substrate_ = 473, 573, and 673 K.

The dependence of the junction resistance on the pillar size was investigated for determining the *RA* values as well as the parasitic resistance (*R*_para_) in the devices. Figure 7a shows the junction resistance (*R*_p_) at the parallel magnetization state between the bottom and top NiMnSb layers as a function of the inverse of the junction area (1/*A*) for the patterned CPP-GMR devices with NiMnSb layers deposited at *T*_substrate_ = 573 K. The dependence of *R*_p_ on 1/*A* is well reproduced by a linear relationship, indicating an excellent electron beam lithography process for controlling the nanopillar sizes. From the linear fitting, *RA* and *R*_para_ were estimated to be 26 ± 1 mΩ·μm^2^ and 0.42 ± 0.05 Ω, respectively for the CPP-GMR junctions. Thus, the CPP-GMR ratio can be deduced using the formula (*R*_ap_-*R*_p_)/(*R*_p_-*R*_para_) where *R*_ap_ represents the junction resistance in an antiparallel magnetization configuration. A CPP-GMR ratio of 8% at RT was achieved in the fully epitaxial NiMnSb/Ag/NiMnSb CPP-GMR junction, as shown in Fig. 7b. A typical pseudo spin-valve MR curve was observed with separated switching fields between the top and bottom NiMnSb layers. Figure 7c shows the *T*_substrate_ dependence of the CPP-GMR ratio and *RA* value for the CPP-GMR junctions. For the samples with NiMnSb layers deposited at 473 K, only a small CPP-GMR ratio was observed because of the low ordering of the NiMnSb layers, as investigated by XRD analysis; on the other hand, the reduction in the CPP-GMR ratio, associated with an increment in the *RA* value to 45 mΩ·μm^2^ for the sample at *T*_substrate_ = 673 K, could be attributed to the increased surface roughness.

**Figure 8:**
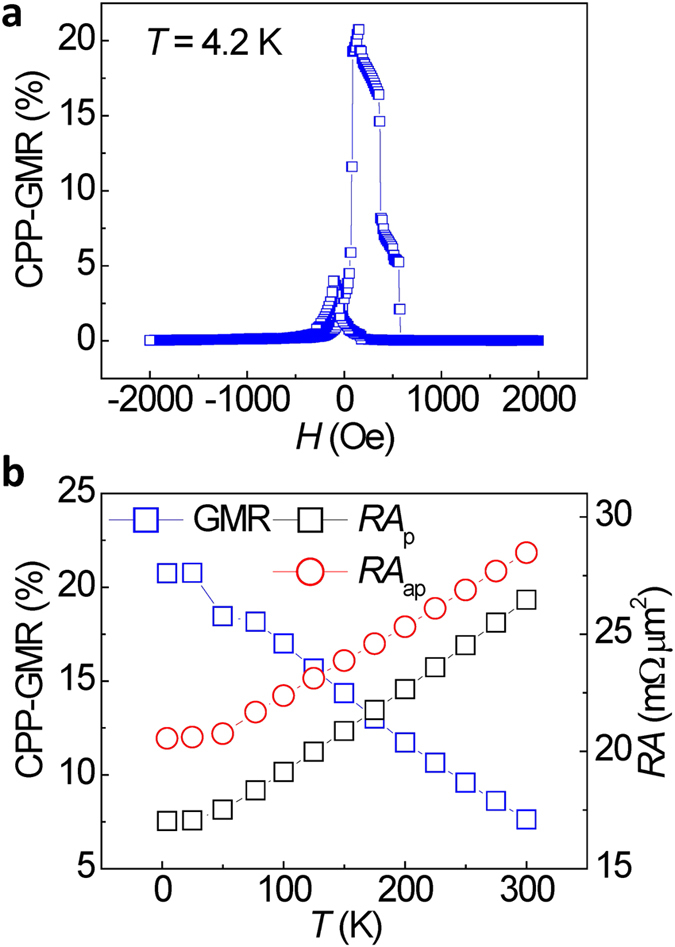
Temperature dependence of magnetic transport properties. (**a**) MR curves for a typical CPP-GMR nanopillar at 4.2 K. (**b)** Dependence of the CPP-GMR ratio on the measuring temperature, and *RA* values at the parallel (*RA*_p_) and antiparallel (*RA*_ap_) magnetic states for the device. NiMnSb layers in the GMR stack were deposited at *T*_substrate_ = 573 K.

Figure 8 shows the dependence of the CPP-GMR ratio on measuring temperature and the *RA* values for a NiMnSb/Ag/NiMnSb CPP-GMR device in which the NiMnSb layers were deposited at *T*_substrate_ = 573 K. A CPP-GMR ratio of 21% was achieved at 4.2 K, as shown in Fig. 8a. The CPP-GMR ratio is enhanced to more than double, which could be attributed to the excellent ordering structure of NiMnSb and the Ag spacer achieved in this study. Moreover, at 4.2 K, an imperfect antiparallel state and antisymmetric feature were observed in the MR loops, indicating an enhanced magnetic coupling between the bottom and the top NiMnSb layers at low temperature^29^. As seen in Fig. 8b, the CPP-GMR ratio monotonically increases with decrease in the measuring temperature, which is different from the anomalous temperature dependence of the CPP-GMR ratio at low temperatures (<80 K) observed in the Co_2_MnSi/Ag/Co_2_MnSi system due to Mn inter-diffusion^30^. This could be indicative that our NiMnSb/Ag/NiMnSb structure is free from Mn inter-diffusion. In contrast to the CPP-GMR ratio, the *RA* values at both the parallel (*RA*_p_) and antiparallel (*RA*_ap_) magnetic states decrease with decreasing temperature.

## Discussion

Well-grown NiMnSb films are of particular significance for maintaining the half metallic property as well as reducing the spin-flip scattering at the NiMnSb/Ag interface for successful demonstration of CPP-GMR devices. Fully (001)-oriented epitaxial NiMnSb films were achieved on Cr/Ag-buffered MgO (001) substrates by optimizing the *T*_substrate_. The optimum condition for the preparation of an epitaxial NiMnSb alloy film with a high magnetization and a very flat surface was achieved by investigating the magnetic properties and the surface morphology. We also deposited NiMnSb films on MgO(001) substrates directly by varying the *T*_substrate_; in this case, only polycrystalline films with multi-phases were observed. These indicate that not only deposition conditions but also well-designed buffer layers are indispensable for the engineering of half-Heusler NiMnSb alloy films with an ideal ordered structure and epitaxial growth.

**Figure 9:**
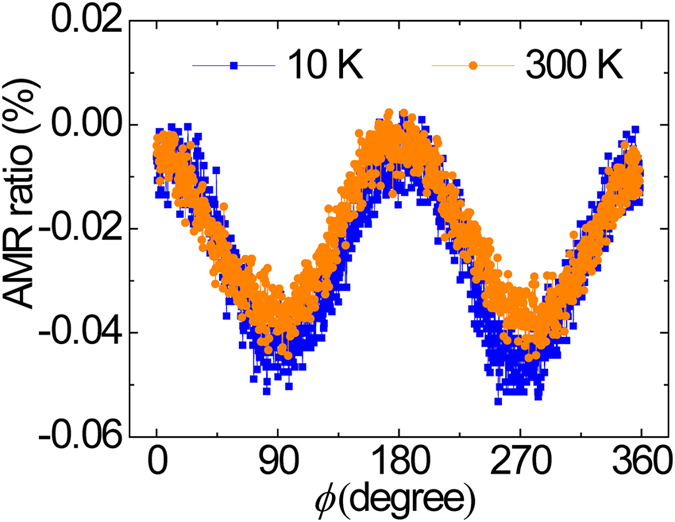
AMR ratio dependence on the in-plane relative angle *ϕ* for a typical NiMnSb single film used in CPP-GMR devices. The measurement was performed at 300 K and 10 K. *ϕ* = 0° (90°) represents that magnetization is normal (parallel) to the measuring current.

Epitaxial CPP-GMR devices with NiMnSb/Ag/NiMnSb multilayers were fabricated and a RT CPP-GMR ratio of 8% was successfully achieved. Study of the spin-dependent transport properties as a function of the measuring temperature revealed a monotonic increase in the CPP-GMR ratio with decreasing temperature. A significant improvement in the CPP-GMR ratio (21%) at low temperatures was achieved. The first observation of RT CPP-GMR in the *C*1_b_-type half-Heusler alloy-based CPP-GMR devices indicates that the composition, structure, and magnetic properties of the NiMnSb alloys and the CPP-GMR stacks are well optimized. According to the Valet–Fert model^31^, the CPP-GMR ratio is proportional to 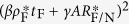 in the limit where spin-diffusion lengths of electrons are much longer than the layer thicknesses. Here, *β* and *γ* are the coefficients of bulk and interfacial spin asymmetry, respectively; *t*_F_ is the thickness of the ferromagnetic layer; 
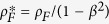
 and 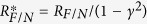 represent the resistivity of the ferromagnetic layer (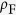) and the resistance at the ferromagnetic/nonmagnetic interface (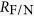) including spin asymmetries, respectively. The increase in the CPP-GMR ratio with decreasing temperature could result from the increase in *β* and *γ* because of high spin polarization at low temperatures owing to the suppression of the thermal excitation of electrons to the bandgap. Furthermore, we measured AMR effect in a typical epitaxial NiMnSb single film utilized in the CPP-GMR devices since AMR ratio was known to be a fingerprint of half-metallicity^32^. Figure 9 shows the dependence of AMR ratio on the in-plane relative angle *ϕ* in the typical NiMnSb single film where *ϕ* = 0° (90°) represents that magnetization is normal (parallel) to the measuring current. The negative AMR ratio was observed which indicates the half metallicity of the film. Especially, the discrepancy of AMR amplitudes between 300 K and 10 K is much smaller than that of Co_2_MnSi full-Heusler alloy films^33^, indicating robust bulk half metallicity against thermal fluctuation in the half-Heusler NiMnSb films. The modest CPP-GMR ratios with relatively large measuring temperature dependence could be attributed to interface effects between NiMnSb and Ag. Additional investigations to improve the interface properties would be required to further increase the CPP-GMR ratio.

Although the modest MR values were shown at present, this pioneering work will stimulate future studies for achieving a higher MR value. This work indicates that the NiMnSb half-Heusler alloy could be a potentially promising material for spintronic applications because of the physical advantages of the half-Heulser compound material, and provides a pathway for engineering a new class of ordered alloy materials with particular emphasis on spintronics.

## Methods

All multilayer stacks were deposited by an ultrahigh vacuum magnetron sputtering system, with a base pressure lower than 1 × 10^−7^ Pa. Prior to depositing NiMnSb films, the bilayers of Cr(20 nm)/Ag(40 nm) were deposited at RT on MgO(001) single-crystalline substrates, where the 20-nm-thick Cr layer was *in*-*situ* post-annealed at 973 K for 1 h. NiMnSb layers were deposited at varied *T*_substrate_ from RT to 773 K by a co-sputtering method using Ni and MnSb targets, and the composition of the deposited NiMnSb films was confirmed by ICP analysis. The structural properties of NiMnSb films on Cr/Ag buffers were characterized by out-of-plane (2*θ*-scan) XRD with Cu *Kα* radiation (λ = 0.15418 nm) and a two-dimensional detector. Surface morphology of NiMnSb half-Heusler alloy films as a function of *T*_substrate_ was investigated by AFM with a scan area of 1 × 1 μm^2^. Magnetization hysteresis loops were measured at RT using a vibrating sample magnetometer. The CPP-GMR multilayer films were nano-fabricated into rectangular pillars with junction sizes ranging from 50 × 100 nm^2^ to 400 × 800 nm^2^ by electron beam lithography and a lift-off technique, while the top and bottom electrical pads were patterned by conventional UV lithography. Magneto-electrical transport properties were measured at RT and low temperatures using a four-probe method in a physical property measurement system.

## Additional Information

**How to cite this article**: Wen, Z. *et al.* Fully epitaxial *C*1_b_-type NiMnSb half-Heusler alloy films for current-perpendicular-to-plane giant magnetoresistance devices with a Ag spacer. *Sci. Rep.* **5**, 18387; doi: 10.1038/srep18387 (2015).

## Supplementary Material

Supporting Information

## Figures and Tables

**Figure 1:**
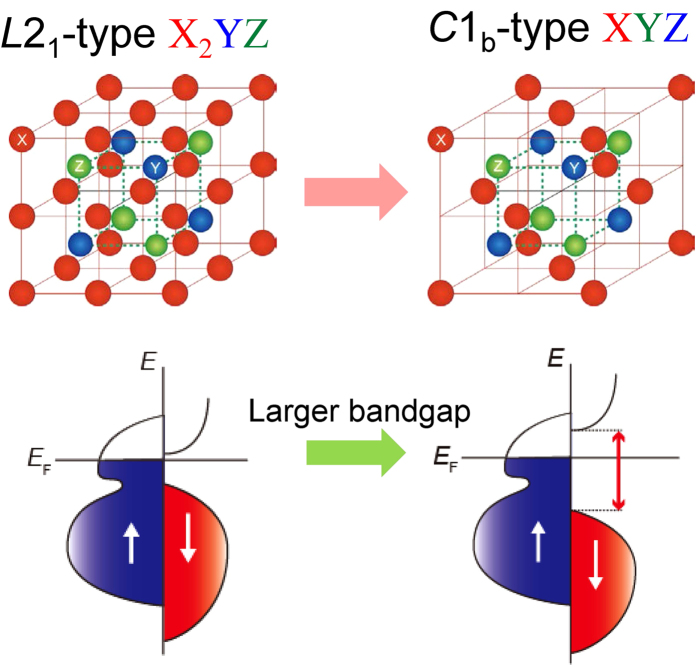
Illustrations of the crystalline cell structures with atom occupancy and electronic band structure near the Fermi level for *L*2_1_-type full-Heusler and *C*1_b_-type half-Heusler alloys.
