# Supplementary material for: Fully epitaxial C1b-type NiMnSb half-Heusler alloy films for current-perpendicular-to-plane giant magnetoresistance devices with a Ag spacer
Source: Sci Rep. 2015 Dec 17;5:18387. doi: 10.1038/srep18387 (PMC4682095; doi:10.1038/srep18387)
Supplement: Supporting Information [file srep18387-s1.doc]

**Supplementary Information**

Fully epitaxial *C*1_b_-type NiMnSb half-Heusler alloys for current-perpendicular-to-plane giant magnetoresistance devices with a Ag spacer

Zhenchao Wen*, Takahide Kubota, Tatsuya Yamamoto & Koki Takanashi

Institute for Materials Research, Tohoku University, Sendai 980-8577, Japan

*E-mail: Wen.Zhenchao@imr.tohoku.ac.jp


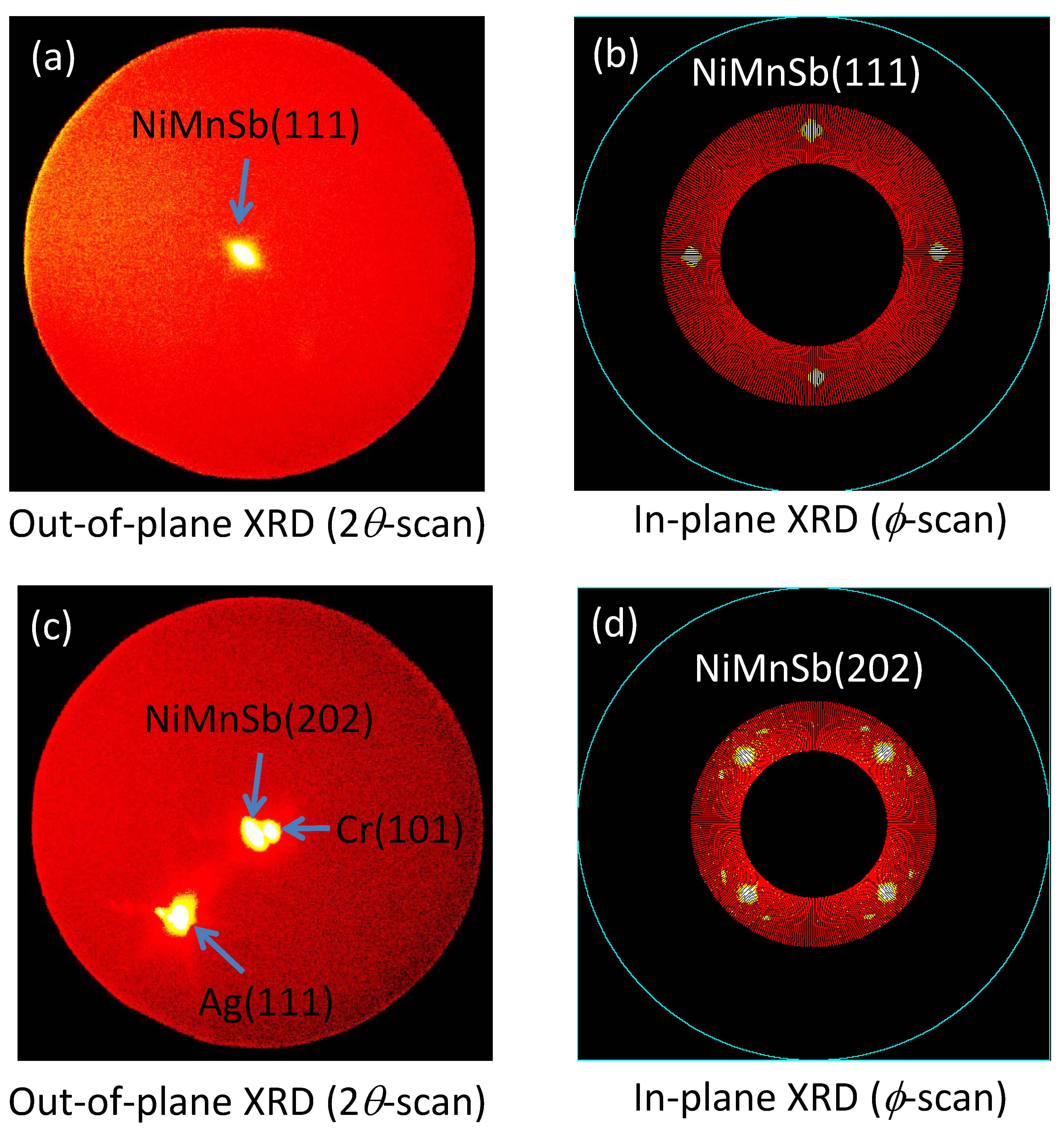


Figure S1. Two dimensional out-of-plane (2*θ*-scan) and in-plane (*φ*-scan) XRD patterns for 50-nm-thick NiMnSb half-Heusler alloy films deposited at *T*_substrate_ = 573 K on Cr(20 nm)/Ag(40 nm)-buffered MgO(001) single crystalline substrates. Clear (a, b) (111) and (c, d) (202) peaks are observed on tilting the sample plane to *χ* = 54.7° and 45°, indicating the realization of the *C*1_b_ structure. The 4-fold peaks observed in the in-plane (*ϕ*-scan) XRD pattern demonstrate that the NiMnSb film has a 4-fold symmetry and fully epitaxial growth in the engineered structure of MgO(001)-substrate//Cr(20)/Ag(40)/NiMnSb(50) (unit: nm).


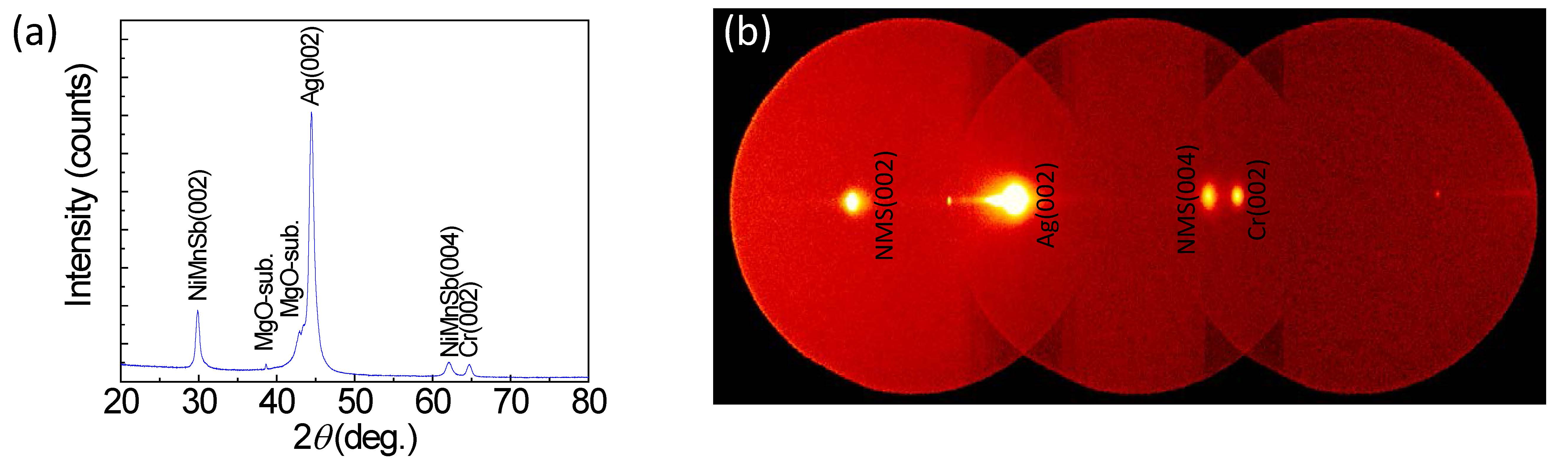


Figure S2. (a) zero and (b) two dimensional out-of-plane XRD patterns for a whole GMR stack with the structure of MgO(001)//Cr(20)/Ag(40)/NiMnSb(20)/Ag(5)/NiMnSb(7)/Ag(2)/Au(7) (unit: nm). Only (001)-oriented peaks can be observed in the entire GMR stack, indicating the uniformity in structure between the bottom and top NiMnSb layers since both of them are deposited upon the Ag(001) layer under the same conditions.
